# Outcomes of Two-Stage Total Hip Replacement for Infected Hemiarthroplasty

**DOI:** 10.7759/cureus.108968

**Published:** 2026-05-16

**Authors:** Amit Kapoor, Nazmee Kabir

**Affiliations:** 1 Orthopaedics, Evercare Hospital, Dhaka, BGD

**Keywords:** infected hemiarthroplasty, infection, periprosthetic joint infection, reoperation, total hip arthroplasty

## Abstract

Infection following hemiarthroplasty of the hip is a devastating complication associated with significant morbidity. While two-stage revision total hip replacement (THR) is the gold standard for chronic periprosthetic joint infection (PJI) in total hip arthroplasty, literature focusing on infected hemiarthroplasty remains limited. This study evaluates the clinical, functional, and radiological outcomes of two-stage revision THR in patients with infected hemiarthroplasty. This retrospective level IV study, conducted in a single center, included 23 patients with confirmed PJI following hemiarthroplasty who underwent two-stage revision THR between January 2015 and January 2024 with a minimum follow-up of two years. Infection was diagnosed using the updated 2018 Musculoskeletal Infection Society (MSIS) criteria. The first stage involved implant removal, debridement, and placement of an antibiotic-loaded cement spacer, followed by targeted antibiotic therapy. Reimplantation was performed after five to seven weeks. Functional outcomes were assessed using the Harris hip score (HHS), and radiological evaluation assessed implant stability. Statistical analysis was performed using a paired t-test. The cohort included 14 females and 9 males, with a mean age of 65.96 years. The mean HHS improved significantly from 26.65 preoperatively to 80.52 postoperatively (p < 0.0001). Infection was eradicated in 95.7% of patients, with one case (4.3%) of recurrent infection. Additionally, 52% of the patients were walking without support, 35% of the patients were using one stick for taking long walks, and 13% of patients always required stick support in the last follow-up. No dislocations, aseptic loosening, or implant migration were observed. There was one intraoperative periprosthetic fracture, which was successfully managed. Two-stage revision THR was associated with high infection-control rates and functional improvement in this series of infected hemiarthroplasty patients and remains a reliable treatment strategy.

## Introduction

Hemiarthroplasty is a very commonly performed procedure for low-demand elderly patients with a fracture of the neck of the femur [1]. Though this procedure was also performed previously for cases of avascular necrosis (AVN) of the femoral head [2], this has gone out of favor due to its inferior results compared to total hip arthroplasty [3]. However, we still encounter cases having hemiarthroplasty for AVN in this part of the subcontinent. With the increasing incidence of femoral neck fractures, the rate of hemiarthroplasty is also increasing [1].

Failure of hemiarthroplasty due to infection is one of the most devastating complications and poses a complex clinical problem requiring meticulous evaluation and management [4]. Periprosthetic joint infection after hemiarthroplasty has been reported in up to 1.74% cases [5]. Infection in a hemiarthroplasty patient with osteoporosis further enhances acetabular articular damage and causes stem loosening [6].

The two-stage revision has been accepted as the preferred strategy for chronic periprosthetic joint infections (PJI) in cases of total hip arthroplasty [7,8] and has reported an infection eradication rate of 85%-90% depending on host factors, virulence/resistance of the organism, and the surgical technique. However, the basic surgical principle remains the same. The first stage involves extensive debridement, implant removal, and placement of an antibiotic spacer, and the second stage is to implant the total hip prosthesis [7,8].

There are multiple reports of conversion of failed hemiarthroplasty to total hip replacement (THR) [9-12]. There are also reports of outcomes of revision THR for infected total hip arthroplasty [13-15], but there remains comparatively limited literature focusing specifically on infected hemiarthroplasty. The treatment of this subgroup presents additional technical challenges, including bone loss, altered anatomy, and the need for careful reconstruction. Our study concerns this specific subgroup of affected individuals with hemiarthroplasty who were debilitated due to infection.

Therefore, this study aims to evaluate the clinical and functional outcomes of two-stage revision THR in patients with infected hemiarthroplasty, with primary emphasis on infection eradication, and secondarily on functional recovery and complication rates.

## Materials and methods

The present study is a retrospective level 4 study design. We analysed the data of the patients in our extensive hospital management system (HMS) and picture archiving and communication system (PACS). Inclusion criteria were confirmed prosthetic infection after a hemiarthroplasty procedure, where a minimum follow-up of two years was available. The criteria for confirmed prosthetic infection were in accordance with the updated 2018 criteria defined by the Musculoskeletal Infection Society (MSIS) [16,17].

From January 2015 to January 2024, 23 patients met the inclusion criteria and had undergone two-stage revision THR of failed infected hemiarthroplasty. Patients with prosthesis failure for causes other than PJI were excluded from the study.

Patient records were analyzed for demographic data, the index surgery timing and details, the patient symptoms at presentation, their functional status preoperatively, and the time between the primary and the first stage of the revision surgery. Functional outcome was assessed by comparing the Harris Hip Score [18] preoperatively and postoperatively at final follow-up.

Any persisting sinus and complications, both intraoperative and postoperative, were also noted. Radiographic analysis preoperatively included assessing the acetabular side. Acetabular erosion was recorded according to the study by Baker et al.: Grade 0 for no erosion, Grade 1 for articular cartilage narrowing without bony erosion, Grade 2 for acetabular bony erosion with early migration of the femoral head prosthesis, and Grade 3 for protrusio acetabuli [19].

Follow-up radiographic images were also assessed from PACS. This involved the pelvis with both hips, AP views during each follow-up visit. The immediate postoperative radiographs were compared with the last follow-up radiographs. Note was made for evidence of any acetabular cup migration vertically or horizontally as per Nunn et al.'s criteria [20] and any subsidence in serial radiographs [21].

The follow-up period was calculated from the date of the second-stage surgery to the date of the final follow-up.

Data were statistically analyzed. Descriptive statistics were used for continuous and categorical variables. The differences between paired measurements were approximately normally distributed; thus, to find the statistical difference between the preoperative and postoperative Harris hip scores (HHS), the paired t-test was used, and p value of less than 0.05 was considered significant.

Surgical technique

First-Stage Surgery

All procedures were performed in the lateral decubitus position through a posterolateral approach. The rotators were cut from their femoral attachments, and the hemiarthroplasty prosthesis was dislocated posteriorly. Then, the prosthesis was removed. The prosthesis came out easily via the use of flexible osteotomes, or Kirschner wires, shooting between the bone implant interface and hammering. None required osteotomy.

**Figure 1:**
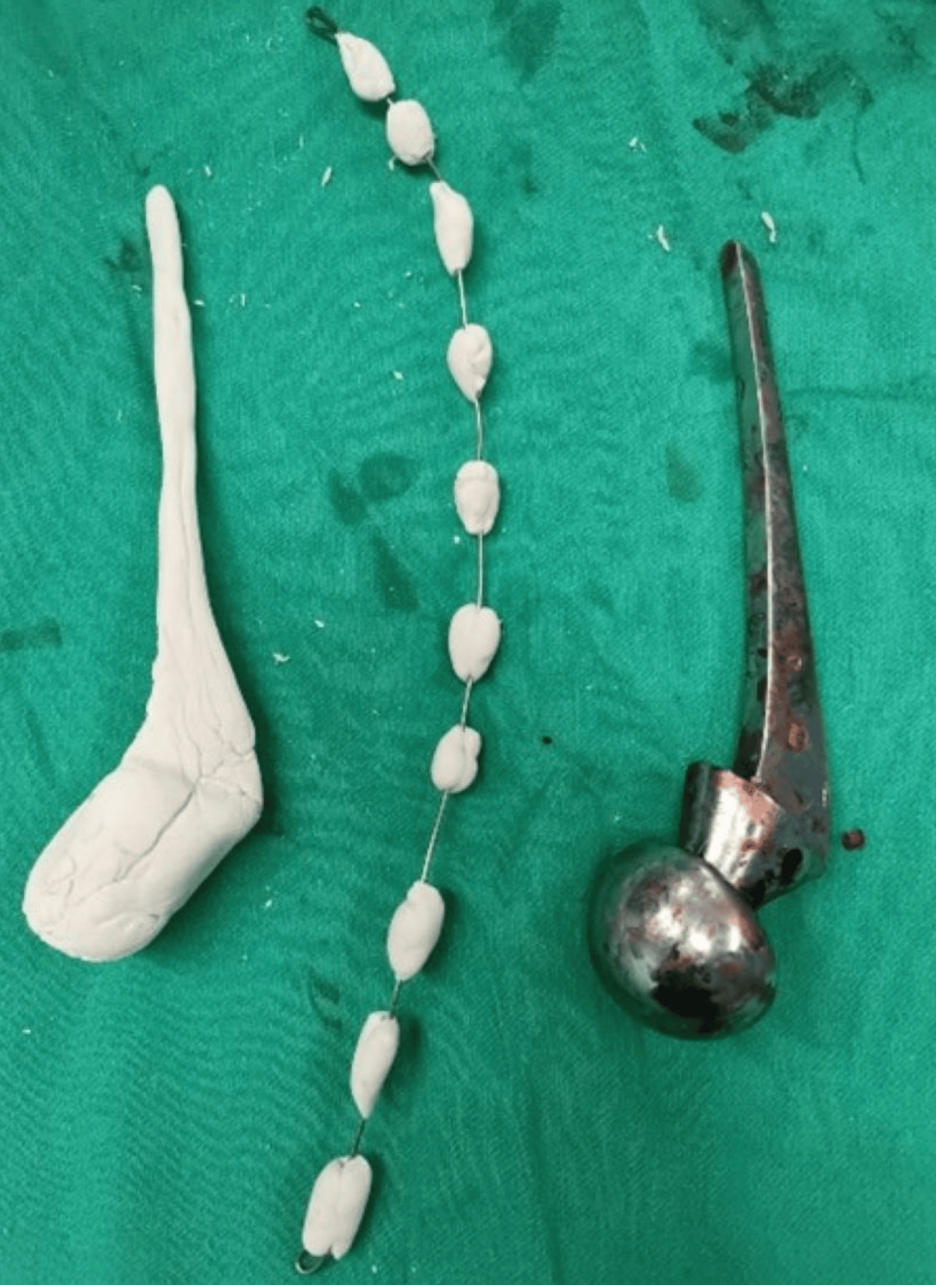
(From left to right) Antibiotic loaded cement spacer prepared perioperatively using bent K-wires, antibiotic cement beads, and extracted Austin Moore prosthesis from infected hemiarthroplasty

**Figure 2:**
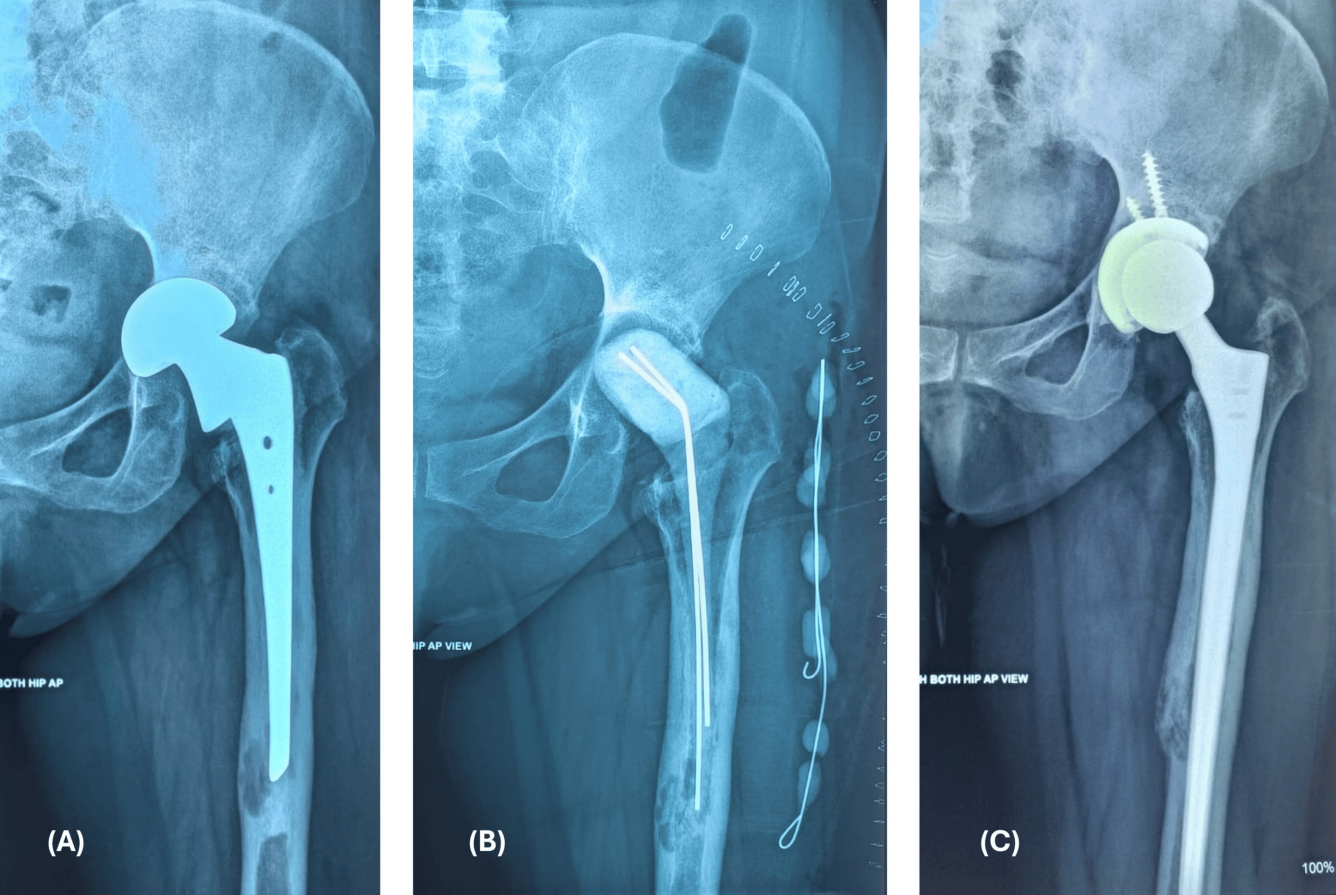
Radiology series of a 59-year-old female (A) Preoperative X-ray with Grade 3 acetabular erosion, (B) X-ray after first-stage surgery with an antibiotic cement spacer and beads, and (C) X-ray at final follow-up with total hip replacement (THR) prosthesis and no signs of loosening.

**Figure 3:**
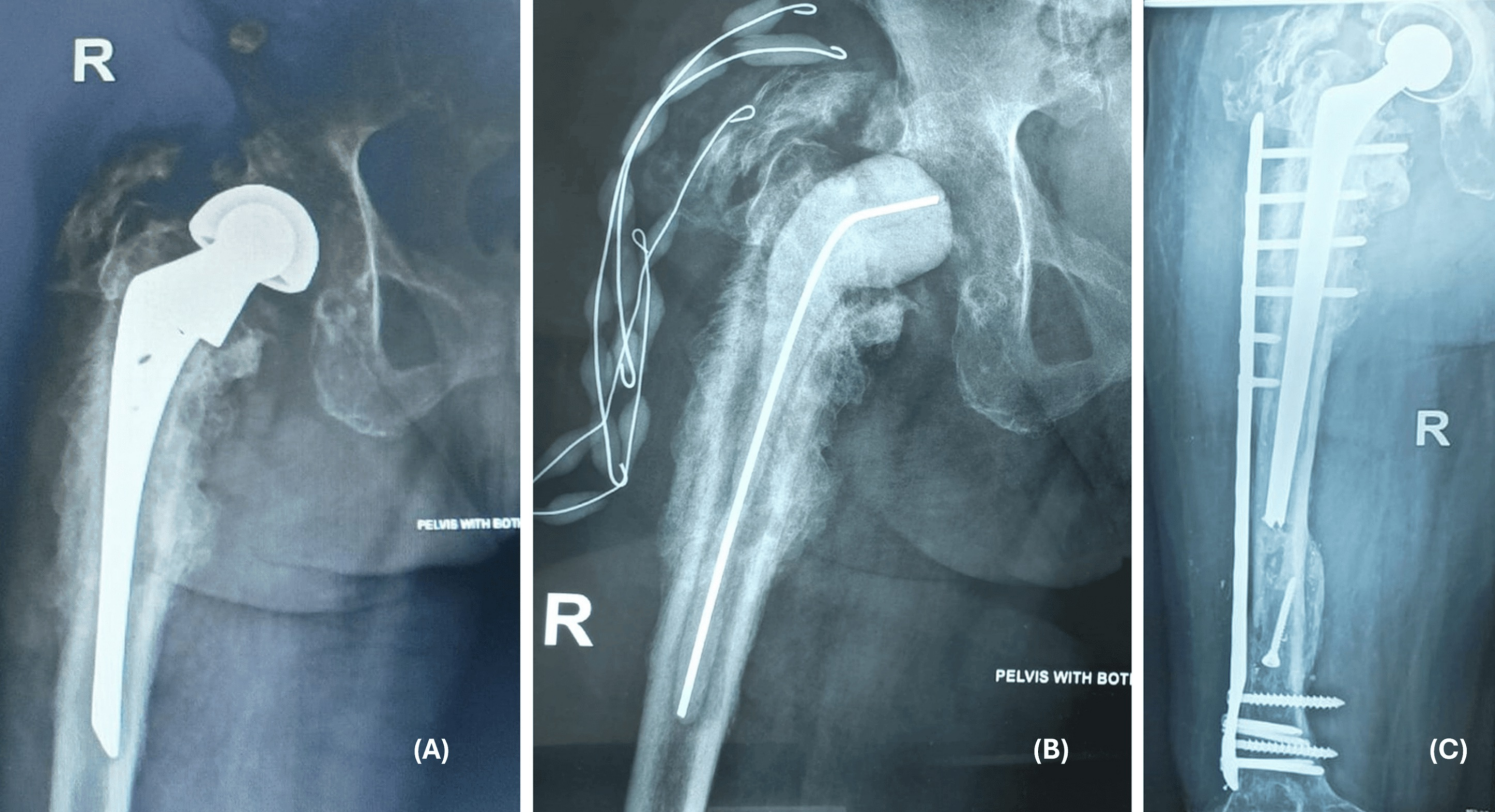
Radiology series of a 74-year-old female with heterotrophic ossification (A) Preoperative X-ray with Grade 2 acetabular erosion, (B) X-ray after first-stage surgery with an antibiotic cement spacer and beads, and (C) X-ray at final follow-up with total hip replacement (THR) prosthesis, also showing the plate and screws, which were used to manage the intraoperative fracture.

All the macroscopic necrotic tissue was debrided. Samples from the necrotic tissue were sent for histopathology and cultures. The intramedullary canal at the distal end of the prosthesis was opened with drill bits. In cases with cement, the cement was removed using osteotomes, a chisel, and drills. The medullary cavity was reamed with hand reamers to remove all debris. Then, copious irrigation of the medullary cavity and acetabulum was done with a minimum of 3 L of 5% povidone iodine, followed by irrigation with a minimum of 3 L of normal saline mixed with gentamycin and vancomycin. Additionally, 500 mg of vancomycin and 80 mg of gentamycin were added to each liter of normal saline. Subsequently, an antibiotic cement spacer was inserted in place of the previous prosthesis (Figure 1, Figure 2B, Figure 3B). Short rotators and capsule were repaired, and antibiotic cement beads were placed around the joint before closing the wound. A cement spacer was made using the Gentamicin bone cement, in which 8 g vancomycin was added, or 4 g vancomycin with 4.5 g of piperacillin-tazobactam was mixed, depending upon the culture reports preoperatively. The latter was used in four of the cases of *Pseudomonas*-positive cultures sensitive to piperacillin-tazobactam. The patients were discharged with antibiotics according to culture and sensitivity reports for patients with positive cultures. In case of patients with negative culture reports, antibiotics were prescribed after consulting with microbiologists. Intravenous antibiotics were prescribed for three weeks, followed by oral antibiotics for two to three weeks till the second-stage surgery. There was no antibiotic holiday.

Second-Stage Surgery

Second-stage surgery was performed after around six weeks (five to seven weeks) from the first stage. By the same approach used in the first stage, all cement beads and the cement spacer were removed, followed by repeated debridement and thorough lavage. A wound swab was sent for culture and sensitivity. The use of implant components for total hip arthroplasty (Figure 2C, Figure 3C) depended on the bone stock, extent of osteolysis, endosteal sclerosis, osteoporosis, and the amount of acetabular protrusion. In one patient, there was a periprosthetic fracture during insertion of the cemented long stem, which required cutting of the tip of the stem and fixation with a plate and screws (Figure 3C).

## Results

Out of the 23 patients, there were 14 women and 9 men, with an average age of 65.96 years, ranging from 49 to 79. Time elapsed since index surgery varied from 4 months to 16 months (mean of nine months).

Severe groin pain was the most predominant complaint, with sinus and discharge. Specifically, 100% of the patients had discharging sinus, which is a major criterion, according to the MSIS criteria [16], 73.9% had positive culture reports, and 26.1% of them showed no growth in cultures (Table 1). Eighteen out of 23 of them had raised erythrocyte sedimentation rate (ESR) and C-reactive protein (CRP) levels. ESR ranged from 8 to 85 mm in the first hour, with a mean of 47 mm in the first hour, and CRP ranged from 0.2 mg/dL to 8.2 mg/dL, with an average of 3.33 mg/dL.

**Table 1:** Demographic parameters and preoperative status for patients undergoing THR for infected hemiarthroplasty THR: total hip replacement; AVN: avascular necrosis

Sl. No	Variable	Range	
1	Age	49-79 years (Mean: 65.9)	
Sl. No	Variable	Frequency	(%)
2	Sex		
	Male	9	39.13%
	Female	14	60.87%
3	Presenting Symptoms (Preoperative)		
	Pain in the groin or thigh	23	100%
	Sinus	23	100%
4	Ambulation		
	Bedridden	3	13.04%
	Inside house ambulation	15	65.22%
	Ambulatory outside the house	5	21.74%
5	Index Procedure Diagnosis		
	Fracture of the neck of the femur	22	95.65%
	AVN	1	4.35%
6	Index Procedure Fixation		
	Uncemented	17	73.91%
	Cemented	6	26.09%
7	Cultures		
	Positive Cultures		
	MRSA	9	39.13%
	Pseudomonas	4	17.39%
	Escherichia coli	2	8.70%
	Staphylococcus haemolyticus	2	8.70%
	Negative Cultures	6	26.09%
8	Preoperative Acetabular Erosion		
	Grade 0	5	21.74%
	Grade 1	6	26.09%
	Grade 2	9	39.13%
	Grade 3	3	13.04%

The mean preoperative HHS was 26.65 (range: 11-45), which significantly increased to a mean postoperative HHS of 80.52 (range: 61-93). This improvement was found to be highly statistically significant (paired t-test, t = 24.9, df = 22, p < 0.0001), indicating a substantial postoperative functional gain (Tables 2-3).

**Table 2:** Preoperative and postoperative HHS HHS: Harris hip score

HHS (preop)	Frequency	HHS (postop)	Frequency
Less than 20	6	Less than 70	4
20-29	11	70-79	4
30 and above	6	80 and above	15
Total	23	Total	23

**Table 3:** Paired t-test results HHS: Harris hip score

Variable	Mean ± SD	Mean Difference (95% CI)	t-value	df	p-value
Preoperative (HHS)	26.65 ± 10.86				
Postoperative (HHS)	80.52 ± 8.89	53.87 (49.4-58.3)	24.9	22	< 0.0001

**Figure 4:**
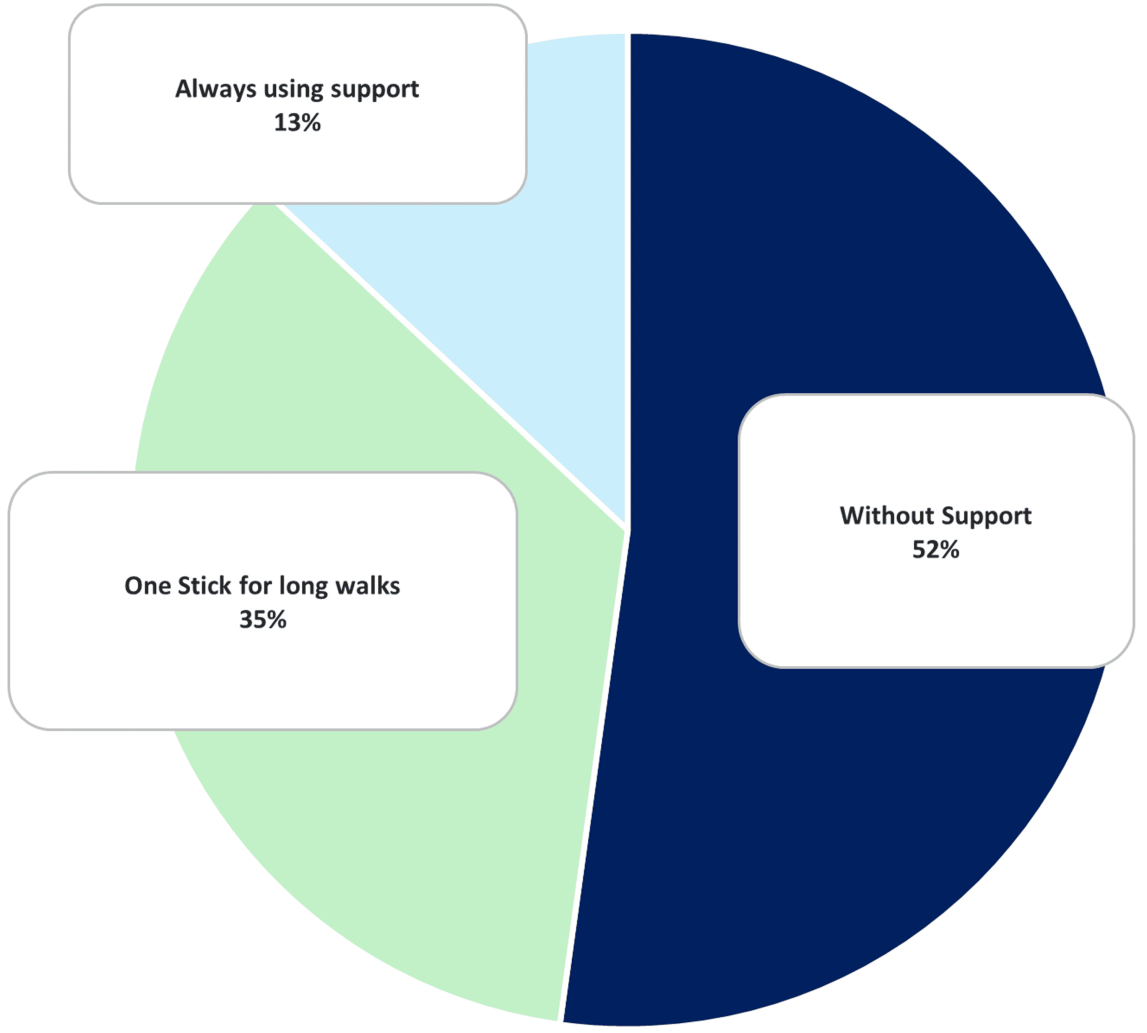
Ambulatory status of the patients at the final follow-up

At the final follow-up, 52% (12) of the patients were walking without support, 35% (8) of the patients were using one stick for taking long walks, and 13% (3) of the patients always required stick support (Figure 4).

All the culture and sensitivity reports from the second-stage surgery came back negative for bacterial growth. There was no postoperative dislocation in any of the patients till the final follow-up. No patient underwent further revision surgeries. In radiological evaluation, there was no evidence of loosening, in final follow-up with respect to progressive radiolucent lines in DeLee or Gruen zones or subsidence of stem or migration of the acetabular cup [20,21].

Out of the 23 patients, only one patient had persistence of infection, which is 4.3%. She developed a discharging sinus four weeks postoperatively and needed to continue antibiotic suppression therapy. The discharging sinus was persistent, appearing at intervals and remaining till the last follow-up at two years; the patient is ambulating with a stick, and her HHS is 64, which was included in the postoperative mean HHS. No other patient developed any sign of persistent or recurrent infection.

In one patient, there was a perioperative periprosthetic fracture at the distal end of the long-cemented stem while inserting the stem. The tip of the stem had protruded and breached the cortex, causing a fracture, and so was cut, and the fracture was fixed with plate osteosynthesis. The patient had heterotrophic ossification preoperatively (Figure 3). The patient's HHS improved from 15 to 61 postoperatively.

## Discussion

Management of PJI following hemiarthroplasty remains a complex and demanding clinical problem, particularly in elderly and comorbid patients. The present study demonstrates that two-stage revision THR provides a reliable and effective treatment option in this subgroup, achieving a high rate of infection eradication alongside significant functional improvement.

In this series, the infection eradication rate was 95.7%, with only one patient (4.3%) demonstrating persistent infection at the final follow-up. This finding is consistent with previously reported success rates of approximately 85-90% for two-stage revision in infected hip arthroplasty [15]. Several studies have reinforced the role of two-stage revision as the gold standard for chronic PJI, particularly in the presence of virulent organisms, poor soft tissue conditions, or unknown pathogens [7,8]. The high eradication rate observed in our cohort supports this principle and further highlights the effectiveness of thorough debridement, appropriate antibiotic spacer use, and targeted antimicrobial therapy, particularly in cases of infected hemiarthroplasty.

Persistent infection rates in a study by Liukkonen et al., where they have performed single-stage revision, DAIR (debridement, antibiotics, and implant retention), and two-stage revisions, have been reported to be higher (26.6%) when compared to the present study [22]. DAIR for infected hemiarthroplasty patients has yielded very poor results, especially in methicillin-resistant *Staphylococcus aureus *(MRSA)* *and *Pseudomonas *infections [5]. These reports discourage and are against the use of implant retention for infected hemiarthroplasties.

A notable finding in this study was the predominance of MRSA among culture-positive cases, which was 53% of the total culture-positive cases. This was followed by *Pseudomonas *as the most common pathogen. These culture reports indicated the infection by more drug-resistant strains. Similar trends of majority infections by multidrug-resistant organisms can be seen in other recent studies [5,23].

A unique step in the present study was the use of piperacillin-tazobactam in the antibiotic cement spacer for four selected cases, which were *Pseudomonas*-positive, in which cultures had shown sensitivity to piperacillin and tazobactam. This was used after in vitro studies had shown satisfactory antibiotic elution from cement that maintained satisfactory antibiotic activity after elution [24].

This may hold further significance due to the emergence of multidrug-resistant organisms with unfavorable antibiotic sensitivity profiles. The choice of an antibiotic mixed in cement, which is tailored to the sensitivity pattern, ought to benefit more than other choices. The use of this can be studied further in in vivo studies with larger sample sizes.

Functionally, patients demonstrated a marked improvement in the HHS from a mean of 26.65 preoperatively to 80.52 postoperatively, which was highly statistically significant. This degree of improvement is comparable to previously reported studies, where HHS improvements from approximately 40-50 to 70-90 have been documented following two-stage revision [4,9,15]. Additionally, most patients in our series regained independent or near-independent ambulation, with 86% requiring either no support or only occasional use of a walking aid.

We used a fixed interval between the two stages of the surgery. The second stage was performed between five and seven weeks after the first stage. The only parameter was healed skin with no localized signs of inflammation, without any monitoring of the CRP or ESR levels during this interval. The success in infection eradication in this study, with this protocol, also points out that the second-stage surgery can be carried out within a shorter interval. Though the other studies have used a longer interval between the first and second stages [25,26], this may be achievable with a shorter delay and lesser patient disabling interval time. The shorter time is supported by the fact that the maximum antibiotic concentration and thus the maximum antimicrobial activity is achieved within the first few hours to days, and the tissue antibiotic concentration reduces progressively [27-29].

The ongoing debate between single-stage and two-stage revision strategies is also relevant. While recent literature suggests that single-stage revision in carefully selected cases [11] may offer comparable reinfection rates and advantages such as reduced cost and faster recovery, two-stage revision remains the preferred approach in complex infections. In our cohort, the high prevalence of resistant organisms, presence of discharging sinuses in all patients, and variable microbiological profiles justified the use of a staged approach.

Complication rates in the present study were low. There were no cases of dislocation or aseptic loosening, and only one intraoperative periprosthetic fracture, which was successfully managed. Radiological evaluation at final follow-up demonstrated stable implants. These findings compare favorably with existing literature, where complication rates of dislocation and intraoperative calcar fractures following revision arthroplasty are higher [9,15].

This study has several strengths. It focuses specifically on infected hemiarthroplasty, a relatively underreported subgroup in the literature, and provides mid-term follow-up data with both clinical and functional outcomes. Additionally, all patients were managed using a consistent surgical protocol, allowing for a more uniform assessment of outcomes.

However, certain limitations must be acknowledged. The sample size is relatively small (n=23), and it is a single-center design, which may limit the generalizability of the findings. The study is also retrospective in nature, and there is no comparison group. Furthermore, while mid-term follow-up is adequate, longer-term outcomes would be valuable in assessing implant survivorship and late complications.

## Conclusions

In the present study, two-stage revision THR for infected hemiarthroplasty was associated with a high infection control rate. It also demonstrated significant functional improvement and restoration of ambulatory capacity in the majority of patients. Two-stage revision THR for infected hemiarthroplasty provides good infection control and significant functional improvement, even in a challenging patient population. Despite its inherent complexity and treatment burden, it remains a reliable and effective strategy, particularly in cases involving resistant organisms and compromised local conditions.
